# (Dimethyl­formamide-κ*O*){4,4′,6,6′-tetra­bromo-2,2′-[*o*-phenyl­enebis(nitrilo­methyl­idyne)]diphenolato-κ^4^ *O*,*N*,*N*′,*O*′}copper(II) dimethyl­formamide solvate

**DOI:** 10.1107/S160053680902162X

**Published:** 2009-06-10

**Authors:** Yu Wu, Bin Xie, Li-Ke Zou, Wei-Ping Wu, Lu Lu

**Affiliations:** a College of Chemistry and Pharmaceutical Engineering, Sichuan University of Science and Engineering, Zigong, Sichuan 643000, People’s Republic of China

## Abstract

In the title compound, [Cu(C_20_H_10_Br_4_N_2_O_2_)(C_3_H_7_NO)]·C_3_H_7_NO, the Cu^II^ ion is coordinated by two N atoms and two O atoms from a tetra­dentate Schiff base ligand and the O atom of one dimethyl­formamide ligand in an almost square-pyramidal geometry. The uncoordinated dimethyl­formamide solvent mol­ecule is disordered over two sets of positions with occupancies of 0.741 (4) and 0.259 (4). The crystal packing is stabilized by C—H⋯O inter­actions.

## Related literature

For the preparation of 3,5-dibromo­salicylaldehyde, see: Elzbieta *et al.* (1964 ▶). For a related structure, see: Bei *et al.* (2003 ▶).
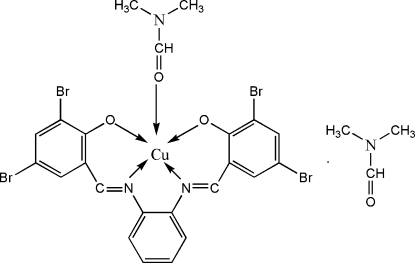


## Experimental

### 

#### Crystal data

[Cu(C_20_H_10_Br_4_N_2_O_2_)(C_3_H_7_NO)]·C_3_H_7_NO
*M* *_r_* = 839.67
Triclinic, 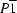

*a* = 7.3742 (10) Å
*b* = 11.9542 (19) Å
*c* = 17.212 (2) Å
α = 94.207 (9)°
β = 100.310 (6)°
γ = 104.117 (5)°
*V* = 1436.8 (3) Å^3^
*Z* = 2
Mo *K*α radiation
μ = 6.36 mm^−1^
*T* = 93 K
0.25 × 0.25 × 0.25 mm

#### Data collection

Rigaku SPIDER diffractometer
Absorption correction: multi-scan (*RAPID-AUTO*; Rigaku, 2004 ▶) *T*_min_ = 0.299, *T*_max_ = 0.299 (expected range = 0.204–0.204)
11906 measured reflections
6330 independent reflections
5305 reflections with *I* > 2σ(*I*)
*R*_int_ = 0.027

#### Refinement

*R*[*F* ^2^ > 2σ(*F* ^2^)] = 0.035
*wR*(*F* ^2^) = 0.063
*S* = 1.02
6330 reflections
392 parameters
8 restraints
H-atom parameters constrained
Δρ_max_ = 0.75 e Å^−3^
Δρ_min_ = −0.52 e Å^−3^

### 

Data collection: *RAPID-AUTO* (Rigaku, 2004 ▶); cell refinement: *RAPID-AUTO*; data reduction: *RAPID-AUTO*; program(s) used to solve structure: *SHELXS97* (Sheldrick, 2008 ▶); program(s) used to refine structure: *SHELXL97* (Sheldrick, 2008 ▶); molecular graphics: *ORTEP-3 for Windows* (Farrugia, 1997 ▶); software used to prepare material for publication: *SHELXL97*.

## Supplementary Material

Crystal structure: contains datablocks global, I. DOI: 10.1107/S160053680902162X/ci2819sup1.cif

Structure factors: contains datablocks I. DOI: 10.1107/S160053680902162X/ci2819Isup2.hkl

Additional supplementary materials:  crystallographic information; 3D view; checkCIF report

## Figures and Tables

**Table 1:** Selected bond lengths (Å)

Cu1—O1	1.910 (2)
Cu1—O2	1.9174 (19)
Cu1—N1	1.954 (2)
Cu1—N2	1.958 (2)
Cu1—O3	2.501 (2)

**Table 2:** Hydrogen-bond geometry (Å, °)

*D*—H⋯*A*	*D*—H	H⋯*A*	*D*⋯*A*	*D*—H⋯*A*
C5—H5⋯O3^i^	0.95	2.47	3.319 (4)	148
C7—H7⋯O3^i^	0.95	2.37	3.243 (4)	153
C14—H14⋯O4^ii^	0.95	2.33	3.226 (14)	156
C22—H22*A*⋯O1	0.98	2.54	3.378 (4)	143

Symmetry codes: (i) 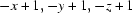; (ii) 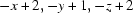.

**Fig. 1.**
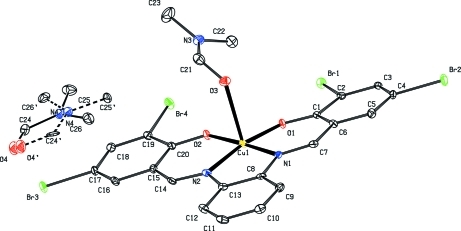
A view of the title complex, showing the atom-labelling scheme. Displacement ellipsoids are drawn at the 30% probability level. H atoms have been omitted for the sake of clarity. Both disorder components of the uncoordinated solvent molecule are shown.

## supplementary crystallographic information

### Comment

The crystal structure and some properties of 1,2-*N*,*N*'-disalicylidenephenylaminato-nickel(II) was previously reported by Bei *et al.* (2003). We report here the synthesis and crystal structure of the title complex, [Cu(C_14_H_8_N_2_O_3_Br_2_)(C_3_H_7_NO)](C_3_H_7_NO).

The contents of the asymmetric unit are shown in Fig.1. The Cu^II^ ion is coordinated by two N atoms and two O atoms from one 4,4',6,6'-tetrabromo-2,2'-[1,2-phenylenebis(nitrilomethylidyne)]diphenolate dianion and one O atom of *N*,*N*-dimethylformamide ligand in a square-pyramidal geometry (Table 1). The crystal packing is stabilized by C—H···O interactions (Table 2).

### Experimental

The title complex was synthesized in two stages. In the first stage, 3,5- dibromosalicylaldehyde was prepared according to Elzbieta *et al.* (1964). Two mole equivalents of 3,5-dibromosalicylaldehyde in ethanol (50 ml) was slowly added to 1,2-phenylenediamine (6 g) in ethanol (100 ml) with continuous stirring. The Schiff base molecule, *viz*. 4,4',6,6'-tetrabromo-2,2'-[1,2-phenylenebis(nitrilomethylidyne)] diphenol, precipitated immediately. In the second stage, the ligand (1 mmol), Cu(OAc)_2_ (1 mmol) and DMF (25 ml) were refluxed for 1 h. The hot solution was filtered and allowed to stand at room temperature undisturbed for about three weeks, resulting in dark green crystals.

### Refinement

The uncoordinated *N*,*N*-dimethylformamide solvent molecule is disordered over two positions with occupancies of 0.741 (4) and 0.259 (4). The N—C(*sp*^3^), N—C(*sp*^2^) and C—O distances in both disorder components were restrained to 1.460 (3), 1.340 (3) and 1.220 (3) Å, respectively. H atoms were positioned geometrically and refined using a riding model, with C—H = 0.95 or 0.98 Å and *U*_iso_(H) = 1.2*U*_eq_(C).

### Crystal data

**Table:**

[Cu(C_20_H_10_Br_4_N_2_O_2_)(C_3_H_7_NO)]·C_3_H_7_NO	*Z* = 2
*M**_r_* = 839.67	*F*(000) = 818
Triclinic, *P*1	*D*_x_ = 1.941 Mg m^−^^3^
Hall symbol: -P 1	Mo *K*α radiation, λ = 0.71073 Å
*a* = 7.3742 (10) Å	Cell parameters from 4531 reflections
*b* = 11.9542 (19) Å	θ = 3.1–27.5°
*c* = 17.212 (2) Å	µ = 6.36 mm^−^^1^
α = 94.207 (9)°	*T* = 93 K
β = 100.310 (6)°	Block, dark green
γ = 104.117 (5)°	0.25 × 0.25 × 0.25 mm
*V* = 1436.8 (3) Å^3^	

### Data collection

**Table:**

Rigaku SPIDER diffractometer	6330 independent reflections
Radiation source: Rotating Anode	5305 reflections with *I* > 2σ(*I*)
graphite	*R*_int_ = 0.027
ω scans	θ_max_ = 27.5°, θ_min_ = 3.1°
Absorption correction: multi-scan (*RAPID-AUTO*; Rigaku, 2004)	*h* = −8→9
*T*_min_ = 0.299, *T*_max_ = 0.299	*k* = −15→15
11906 measured reflections	*l* = −22→22

### Refinement

**Table:**

Refinement on *F*^2^	Primary atom site location: structure-invariant direct methods
Least-squares matrix: full	Secondary atom site location: difference Fourier map
*R*[*F*^2^ > 2σ(*F*^2^)] = 0.035	Hydrogen site location: inferred from neighbouring sites
*wR*(*F*^2^) = 0.063	H-atom parameters constrained
*S* = 1.02	*w* = 1/[σ^2^(*F*_o_^2^) + (0.0247*P*)^2^] where *P* = (*F*_o_^2^ + 2*F*_c_^2^)/3
6330 reflections	(Δ/σ)_max_ = 0.002
392 parameters	Δρ_max_ = 0.75 e Å^−^^3^
8 restraints	Δρ_min_ = −0.52 e Å^−^^3^

### Special details

**Table:**

Geometry. All e.s.d.'s (except the e.s.d. in the dihedral angle between two l.s. planes) are estimated using the full covariance matrix. The cell e.s.d.'s are taken into account individually in the estimation of e.s.d.'s in distances, angles and torsion angles; correlations between e.s.d.'s in cell parameters are only used when they are defined by crystal symmetry. An approximate (isotropic) treatment of cell e.s.d.'s is used for estimating e.s.d.'s involving l.s. planes.
Refinement. Refinement of *F*^2^ against ALL reflections. The weighted *R*-factor *wR* and goodness of fit *S* are based on *F*^2^, conventional *R*-factors *R* are based on *F*, with *F* set to zero for negative *F*^2^. The threshold expression of *F*^2^ > σ(*F*^2^) is used only for calculating *R*-factors(gt) *etc*. and is not relevant to the choice of reflections for refinement. *R*-factors based on *F*^2^ are statistically about twice as large as those based on *F*, and *R*- factors based on ALL data will be even larger.

### Fractional atomic coordinates and isotropic or equivalent isotropic displacement parameters (Å^2^)

**Table:**

	*x*	*y*	*z*	*U*_iso_*/*U*_eq_	Occ. (<1)
Cu1	0.39299 (5)	0.60816 (3)	0.67052 (2)	0.01305 (9)	
Br1	0.12215 (5)	0.88993 (3)	0.535887 (19)	0.01998 (8)	
Br2	−0.00681 (5)	0.60574 (3)	0.244696 (18)	0.02312 (9)	
Br3	0.74637 (5)	0.75127 (3)	1.105720 (18)	0.02390 (9)	
Br4	0.49378 (5)	0.96915 (3)	0.842406 (19)	0.02212 (9)	
O1	0.2620 (3)	0.69347 (17)	0.60103 (11)	0.0141 (5)	
O2	0.4409 (3)	0.73265 (18)	0.75456 (11)	0.0152 (5)	
N1	0.3449 (3)	0.4761 (2)	0.58884 (14)	0.0124 (5)	
N2	0.4835 (4)	0.5038 (2)	0.74155 (14)	0.0142 (6)	
C1	0.2062 (4)	0.6699 (3)	0.52455 (17)	0.0129 (6)	
C2	0.1319 (4)	0.7497 (3)	0.48000 (17)	0.0137 (6)	
C3	0.0698 (4)	0.7320 (3)	0.39909 (17)	0.0150 (7)	
H3	0.0224	0.7885	0.3718	0.018*	
C4	0.0777 (4)	0.6295 (3)	0.35772 (17)	0.0161 (7)	
C5	0.1450 (4)	0.5472 (3)	0.39550 (17)	0.0157 (7)	
H5	0.1484	0.4777	0.3660	0.019*	
C6	0.2099 (4)	0.5664 (3)	0.47917 (17)	0.0125 (6)	
C7	0.2743 (4)	0.4742 (3)	0.51432 (18)	0.0142 (6)	
H7	0.2642	0.4061	0.4799	0.017*	
C8	0.3973 (4)	0.3788 (3)	0.61884 (18)	0.0134 (6)	
C9	0.3827 (4)	0.2740 (3)	0.57325 (19)	0.0172 (7)	
H9	0.3361	0.2643	0.5174	0.021*	
C10	0.4360 (4)	0.1849 (3)	0.60964 (19)	0.0191 (7)	
H10	0.4272	0.1139	0.5788	0.023*	
C11	0.5026 (5)	0.1987 (3)	0.69131 (19)	0.0217 (7)	
H11	0.5367	0.1361	0.7159	0.026*	
C12	0.5201 (4)	0.3013 (3)	0.73727 (19)	0.0184 (7)	
H12	0.5667	0.3097	0.7931	0.022*	
C13	0.4690 (4)	0.3924 (3)	0.70124 (18)	0.0139 (6)	
C14	0.5440 (4)	0.5275 (3)	0.81769 (17)	0.0149 (7)	
H14	0.5803	0.4682	0.8457	0.018*	
C15	0.5606 (4)	0.6358 (3)	0.86300 (17)	0.0147 (7)	
C16	0.6296 (4)	0.6425 (3)	0.94604 (17)	0.0168 (7)	
H16	0.6600	0.5771	0.9680	0.020*	
C17	0.6526 (4)	0.7431 (3)	0.99485 (17)	0.0172 (7)	
C18	0.6111 (4)	0.8407 (3)	0.96363 (18)	0.0174 (7)	
H18	0.6280	0.9104	0.9976	0.021*	
C19	0.5458 (4)	0.8344 (3)	0.88351 (18)	0.0156 (7)	
C20	0.5124 (4)	0.7327 (3)	0.82895 (17)	0.0142 (7)	
O3	0.7162 (3)	0.69177 (19)	0.64181 (13)	0.0218 (5)	
N3	0.8292 (4)	0.8852 (2)	0.68559 (16)	0.0251 (7)	
C21	0.8417 (5)	0.7771 (3)	0.6803 (2)	0.0272 (8)	
H21	0.9556	0.7628	0.7081	0.033*	
C22	0.6627 (5)	0.9150 (3)	0.6398 (2)	0.0278 (8)	
H22A	0.5753	0.8447	0.6082	0.033*	
H22B	0.7052	0.9719	0.6043	0.033*	
H22C	0.5967	0.9483	0.6765	0.033*	
C23	0.9791 (6)	0.9809 (3)	0.7305 (3)	0.0478 (12)	
H23A	1.0806	0.9512	0.7597	0.057*	
H23B	0.9273	1.0233	0.7682	0.057*	
H23C	1.0315	1.0333	0.6941	0.057*	
O4	1.225 (3)	0.6119 (8)	1.0649 (3)	0.0317 (16)	0.741 (4)
C24	1.1892 (7)	0.7023 (4)	1.0473 (2)	0.0281 (12)	0.741 (4)
H24	1.2090	0.7632	1.0891	0.034*	0.741 (4)
N4	1.1242 (16)	0.7216 (4)	0.9731 (2)	0.0216 (12)	0.741 (4)
C25	1.0952 (8)	0.8347 (4)	0.9571 (4)	0.0382 (14)	0.741 (4)
H25A	1.1095	0.8828	1.0075	0.046*	0.741 (4)
H25B	0.9665	0.8243	0.9254	0.046*	0.741 (4)
H25C	1.1900	0.8729	0.9276	0.046*	0.741 (4)
C26	1.0890 (7)	0.6327 (4)	0.9057 (3)	0.0340 (13)	0.741 (4)
H26A	1.1286	0.5649	0.9240	0.041*	0.741 (4)
H26B	1.1622	0.6637	0.8663	0.041*	0.741 (4)
H26C	0.9525	0.6095	0.8815	0.041*	0.741 (4)
O4'	1.229 (8)	0.603 (3)	1.0485 (10)	0.0317 (16)	0.26
C24'	1.1591 (17)	0.6115 (9)	0.9800 (7)	0.018 (3)	0.259 (4)
H24'	1.1272	0.5434	0.9429	0.022*	0.259 (4)
N4'	1.123 (5)	0.7071 (13)	0.9520 (7)	0.0216 (12)	0.26
C25'	1.0462 (17)	0.7099 (12)	0.8680 (6)	0.022 (3)	0.259 (4)
H25D	1.1422	0.7618	0.8453	0.026*	0.259 (4)
H25E	0.9312	0.7383	0.8630	0.026*	0.259 (4)
H25F	1.0138	0.6314	0.8395	0.026*	0.259 (4)
C26'	1.1704 (18)	0.8156 (10)	1.0052 (7)	0.019 (3)	0.259 (4)
H26D	1.0541	0.8407	1.0070	0.023*	0.259 (4)
H26E	1.2613	0.8756	0.9854	0.023*	0.259 (4)
H26F	1.2280	0.8034	1.0587	0.023*	0.259 (4)

### Atomic displacement parameters (Å^2^)

**Table:**

	*U*^11^	*U*^22^	*U*^33^	*U*^12^	*U*^13^	*U*^23^
Cu1	0.0175 (2)	0.01176 (19)	0.01046 (18)	0.00541 (16)	0.00143 (15)	0.00295 (15)
Br1	0.02559 (19)	0.01350 (16)	0.02113 (17)	0.00819 (14)	0.00104 (14)	0.00254 (13)
Br2	0.02115 (18)	0.0359 (2)	0.01140 (16)	0.00857 (15)	−0.00079 (13)	0.00359 (14)
Br3	0.02659 (19)	0.0333 (2)	0.01011 (16)	0.00644 (15)	0.00017 (14)	0.00550 (14)
Br4	0.0337 (2)	0.01807 (17)	0.01618 (16)	0.01086 (15)	0.00296 (14)	0.00318 (13)
O1	0.0191 (12)	0.0135 (11)	0.0094 (10)	0.0063 (9)	−0.0006 (9)	0.0018 (9)
O2	0.0226 (12)	0.0148 (11)	0.0090 (10)	0.0081 (10)	0.0009 (9)	0.0018 (9)
N1	0.0110 (13)	0.0112 (13)	0.0143 (13)	0.0025 (10)	0.0010 (11)	0.0016 (11)
N2	0.0157 (14)	0.0127 (13)	0.0164 (13)	0.0049 (11)	0.0060 (11)	0.0050 (11)
C1	0.0115 (15)	0.0142 (16)	0.0120 (15)	0.0013 (12)	0.0015 (12)	0.0045 (13)
C2	0.0134 (16)	0.0121 (15)	0.0160 (16)	0.0029 (12)	0.0038 (13)	0.0035 (13)
C3	0.0103 (15)	0.0182 (17)	0.0167 (16)	0.0031 (13)	0.0013 (13)	0.0080 (13)
C4	0.0119 (16)	0.0222 (18)	0.0114 (15)	0.0017 (13)	−0.0014 (13)	0.0032 (14)
C5	0.0166 (17)	0.0175 (17)	0.0127 (15)	0.0043 (13)	0.0039 (13)	−0.0017 (13)
C6	0.0146 (16)	0.0131 (16)	0.0118 (15)	0.0057 (13)	0.0043 (13)	0.0027 (13)
C7	0.0143 (16)	0.0127 (16)	0.0160 (16)	0.0014 (13)	0.0070 (13)	0.0017 (13)
C8	0.0093 (15)	0.0114 (15)	0.0209 (16)	0.0021 (12)	0.0058 (13)	0.0060 (13)
C9	0.0149 (16)	0.0156 (16)	0.0202 (17)	0.0039 (13)	0.0013 (14)	0.0027 (14)
C10	0.0188 (17)	0.0125 (16)	0.0267 (18)	0.0047 (14)	0.0064 (15)	0.0015 (14)
C11	0.0241 (19)	0.0177 (17)	0.0294 (19)	0.0095 (14)	0.0117 (15)	0.0127 (15)
C12	0.0197 (17)	0.0226 (18)	0.0181 (17)	0.0098 (14)	0.0088 (14)	0.0086 (14)
C13	0.0145 (16)	0.0113 (15)	0.0179 (16)	0.0041 (12)	0.0064 (13)	0.0031 (13)
C14	0.0146 (16)	0.0183 (17)	0.0144 (15)	0.0055 (13)	0.0049 (13)	0.0094 (13)
C15	0.0122 (16)	0.0202 (17)	0.0120 (15)	0.0049 (13)	0.0017 (13)	0.0029 (13)
C16	0.0147 (16)	0.0237 (18)	0.0150 (16)	0.0085 (14)	0.0028 (13)	0.0101 (14)
C17	0.0158 (17)	0.0257 (18)	0.0085 (15)	0.0039 (14)	0.0001 (13)	0.0027 (14)
C18	0.0152 (17)	0.0217 (18)	0.0151 (16)	0.0047 (14)	0.0036 (13)	0.0003 (14)
C19	0.0168 (17)	0.0161 (16)	0.0159 (16)	0.0066 (13)	0.0042 (13)	0.0049 (13)
C20	0.0113 (16)	0.0193 (17)	0.0127 (15)	0.0041 (13)	0.0034 (12)	0.0037 (13)
O3	0.0247 (13)	0.0146 (12)	0.0243 (13)	0.0014 (10)	0.0073 (11)	−0.0015 (10)
N3	0.0296 (17)	0.0163 (15)	0.0258 (16)	0.0010 (13)	0.0043 (13)	−0.0002 (13)
C21	0.030 (2)	0.029 (2)	0.0271 (19)	0.0131 (17)	0.0084 (17)	0.0096 (17)
C22	0.036 (2)	0.0224 (19)	0.0259 (19)	0.0092 (17)	0.0055 (17)	0.0013 (16)
C23	0.047 (3)	0.023 (2)	0.057 (3)	−0.0038 (19)	−0.012 (2)	−0.001 (2)
O4	0.0336 (19)	0.031 (2)	0.029 (3)	0.0112 (18)	−0.003 (4)	0.011 (2)
C24	0.021 (3)	0.032 (3)	0.027 (3)	0.004 (2)	−0.002 (2)	0.006 (2)
N4	0.0193 (16)	0.021 (2)	0.024 (3)	0.005 (2)	0.001 (4)	0.007 (2)
C25	0.030 (3)	0.035 (3)	0.056 (4)	0.012 (3)	0.014 (3)	0.023 (3)
C26	0.024 (3)	0.049 (4)	0.032 (3)	0.010 (3)	0.008 (2)	0.012 (3)
O4'	0.0336 (19)	0.031 (2)	0.029 (3)	0.0112 (18)	−0.003 (4)	0.011 (2)
C24'	0.013 (6)	0.013 (6)	0.028 (7)	−0.004 (5)	0.007 (6)	0.011 (6)
N4'	0.0193 (16)	0.021 (2)	0.024 (3)	0.005 (2)	0.001 (4)	0.007 (2)
C25'	0.014 (7)	0.037 (8)	0.014 (6)	0.006 (6)	−0.003 (5)	0.016 (6)
C26'	0.013 (7)	0.021 (8)	0.024 (7)	0.005 (6)	0.004 (6)	−0.002 (6)

### Geometric parameters (Å, °)

**Table:**

Cu1—O1	1.910 (2)	C16—C17	1.372 (4)
Cu1—O2	1.9174 (19)	C16—H16	0.95
Cu1—N1	1.954 (2)	C17—C18	1.397 (4)
Cu1—N2	1.958 (2)	C18—C19	1.367 (4)
Cu1—O3	2.501 (2)	C18—H18	0.95
Br1—C2	1.894 (3)	C19—C20	1.423 (4)
Br2—C4	1.911 (3)	O3—C21	1.247 (4)
Br3—C17	1.897 (3)	N3—C21	1.316 (4)
Br4—C19	1.901 (3)	N3—C23	1.441 (4)
O1—C1	1.295 (3)	N3—C22	1.471 (4)
O2—C20	1.294 (3)	C21—H21	0.95
N1—C7	1.291 (4)	C22—H22A	0.98
N1—C8	1.416 (4)	C22—H22B	0.98
N2—C14	1.293 (4)	C22—H22C	0.98
N2—C13	1.426 (4)	C23—H23A	0.98
C1—C2	1.418 (4)	C23—H23B	0.98
C1—C6	1.423 (4)	C23—H23C	0.98
C2—C3	1.369 (4)	O4—C24	1.218 (3)
C3—C4	1.390 (4)	C24—N4	1.340 (3)
C3—H3	0.95	C24—H24	0.95
C4—C5	1.361 (4)	N4—C26	1.456 (3)
C5—C6	1.416 (4)	N4—C25	1.457 (3)
C5—H5	0.95	C25—H25A	0.98
C6—C7	1.434 (4)	C25—H25B	0.98
C7—H7	0.95	C25—H25C	0.98
C8—C9	1.399 (4)	C26—H26A	0.98
C8—C13	1.405 (4)	C26—H26B	0.98
C9—C10	1.377 (4)	C26—H26C	0.98
C9—H9	0.95	O4'—C24'	1.220 (3)
C10—C11	1.387 (4)	C24'—N4'	1.340 (3)
C10—H10	0.95	C24'—H24'	0.95
C11—C12	1.375 (4)	N4'—C25'	1.460 (3)
C11—H11	0.95	N4'—C26'	1.460 (3)
C12—C13	1.389 (4)	C25'—H25D	0.98
C12—H12	0.95	C25'—H25E	0.98
C14—C15	1.428 (4)	C25'—H25F	0.98
C14—H14	0.95	C26'—H26D	0.98
C15—C16	1.418 (4)	C26'—H26E	0.98
C15—C20	1.427 (4)	C26'—H26F	0.98
			
O1—Cu1—O2	89.11 (8)	C16—C15—C14	115.6 (3)
O1—Cu1—N1	93.40 (9)	C20—C15—C14	123.6 (3)
O2—Cu1—N1	177.19 (10)	C17—C16—C15	120.3 (3)
O1—Cu1—N2	170.18 (10)	C17—C16—H16	119.9
O2—Cu1—N2	93.64 (9)	C15—C16—H16	119.9
N1—Cu1—N2	83.67 (10)	C16—C17—C18	120.7 (3)
O1—Cu1—O3	96.00 (9)	C16—C17—Br3	119.8 (2)
O2—Cu1—O3	89.70 (8)	C18—C17—Br3	119.4 (2)
N1—Cu1—O3	91.29 (9)	C19—C18—C17	119.0 (3)
N2—Cu1—O3	93.44 (9)	C19—C18—H18	120.5
C1—O1—Cu1	126.73 (19)	C17—C18—H18	120.5
C20—O2—Cu1	126.9 (2)	C18—C19—C20	124.0 (3)
C7—N1—C8	121.5 (3)	C18—C19—Br4	118.1 (2)
C7—N1—Cu1	125.3 (2)	C20—C19—Br4	117.9 (2)
C8—N1—Cu1	113.22 (19)	O2—C20—C19	119.9 (3)
C14—N2—C13	121.4 (3)	O2—C20—C15	124.9 (3)
C14—N2—Cu1	125.7 (2)	C19—C20—C15	115.2 (3)
C13—N2—Cu1	112.92 (19)	C21—O3—Cu1	126.8 (2)
O1—C1—C2	119.9 (3)	C21—N3—C23	123.2 (3)
O1—C1—C6	125.0 (3)	C21—N3—C22	120.3 (3)
C2—C1—C6	115.1 (3)	C23—N3—C22	116.3 (3)
C3—C2—C1	123.8 (3)	O3—C21—N3	125.4 (3)
C3—C2—Br1	118.3 (2)	O3—C21—H21	117.3
C1—C2—Br1	117.8 (2)	N3—C21—H21	117.3
C2—C3—C4	118.6 (3)	N3—C22—H22A	109.5
C2—C3—H3	120.7	N3—C22—H22B	109.5
C4—C3—H3	120.7	H22A—C22—H22B	109.5
C5—C4—C3	121.8 (3)	N3—C22—H22C	109.5
C5—C4—Br2	119.8 (2)	H22A—C22—H22C	109.5
C3—C4—Br2	118.5 (2)	H22B—C22—H22C	109.5
C4—C5—C6	119.3 (3)	N3—C23—H23A	109.5
C4—C5—H5	120.3	N3—C23—H23B	109.5
C6—C5—H5	120.3	H23A—C23—H23B	109.5
C5—C6—C1	121.4 (3)	N3—C23—H23C	109.5
C5—C6—C7	115.7 (3)	H23A—C23—H23C	109.5
C1—C6—C7	123.0 (3)	H23B—C23—H23C	109.5
N1—C7—C6	125.4 (3)	O4—C24—N4	124.5 (4)
N1—C7—H7	117.3	O4—C24—H24	117.8
C6—C7—H7	117.3	N4—C24—H24	117.8
C9—C8—C13	119.4 (3)	C24—N4—C26	121.2 (4)
C9—C8—N1	125.2 (3)	C24—N4—C25	120.7 (4)
C13—C8—N1	115.3 (3)	C26—N4—C25	118.0 (4)
C10—C9—C8	119.8 (3)	O4'—C24'—N4'	126.4 (14)
C10—C9—H9	120.1	O4'—C24'—H24'	116.8
C8—C9—H9	120.1	N4'—C24'—H24'	116.8
C9—C10—C11	120.1 (3)	C24'—N4'—C25'	122.5 (11)
C9—C10—H10	119.9	C24'—N4'—C26'	119.9 (11)
C11—C10—H10	119.9	C25'—N4'—C26'	117.5 (10)
C12—C11—C10	121.2 (3)	N4'—C25'—H25D	109.5
C12—C11—H11	119.4	N4'—C25'—H25E	109.5
C10—C11—H11	119.4	H25D—C25'—H25E	109.5
C11—C12—C13	119.3 (3)	N4'—C25'—H25F	109.5
C11—C12—H12	120.3	H25D—C25'—H25F	109.5
C13—C12—H12	120.3	H25E—C25'—H25F	109.5
C12—C13—C8	120.1 (3)	N4'—C26'—H26D	109.5
C12—C13—N2	125.0 (3)	N4'—C26'—H26E	109.5
C8—C13—N2	114.8 (3)	H26D—C26'—H26E	109.5
N2—C14—C15	125.1 (3)	N4'—C26'—H26F	109.5
N2—C14—H14	117.4	H26D—C26'—H26F	109.5
C15—C14—H14	117.4	H26E—C26'—H26F	109.5
C16—C15—C20	120.8 (3)		
			
O2—Cu1—O1—C1	169.2 (2)	C8—C9—C10—C11	0.5 (5)
N1—Cu1—O1—C1	−12.0 (2)	C9—C10—C11—C12	−1.2 (5)
O3—Cu1—O1—C1	79.6 (2)	C10—C11—C12—C13	0.4 (5)
O1—Cu1—O2—C20	171.6 (2)	C11—C12—C13—C8	1.0 (5)
N2—Cu1—O2—C20	1.1 (3)	C11—C12—C13—N2	−178.7 (3)
O3—Cu1—O2—C20	−92.4 (2)	C9—C8—C13—C12	−1.6 (5)
O1—Cu1—N1—C7	9.3 (3)	N1—C8—C13—C12	178.3 (3)
N2—Cu1—N1—C7	179.9 (3)	C9—C8—C13—N2	178.1 (3)
O3—Cu1—N1—C7	−86.8 (3)	N1—C8—C13—N2	−1.9 (4)
O1—Cu1—N1—C8	−171.3 (2)	C14—N2—C13—C12	−1.5 (5)
N2—Cu1—N1—C8	−0.7 (2)	Cu1—N2—C13—C12	−179.0 (3)
O3—Cu1—N1—C8	92.6 (2)	C14—N2—C13—C8	178.7 (3)
O2—Cu1—N2—C14	1.5 (3)	Cu1—N2—C13—C8	1.3 (3)
N1—Cu1—N2—C14	−177.6 (3)	C13—N2—C14—C15	−179.1 (3)
O3—Cu1—N2—C14	91.5 (3)	Cu1—N2—C14—C15	−2.0 (4)
O2—Cu1—N2—C13	178.8 (2)	N2—C14—C15—C16	179.7 (3)
N1—Cu1—N2—C13	−0.3 (2)	N2—C14—C15—C20	−0.4 (5)
O3—Cu1—N2—C13	−91.2 (2)	C20—C15—C16—C17	−0.6 (5)
Cu1—O1—C1—C2	−172.1 (2)	C14—C15—C16—C17	179.3 (3)
Cu1—O1—C1—C6	8.9 (4)	C15—C16—C17—C18	−0.9 (5)
O1—C1—C2—C3	179.9 (3)	C15—C16—C17—Br3	−179.6 (2)
C6—C1—C2—C3	−1.0 (5)	C16—C17—C18—C19	0.4 (5)
O1—C1—C2—Br1	1.0 (4)	Br3—C17—C18—C19	179.2 (2)
C6—C1—C2—Br1	−179.9 (2)	C17—C18—C19—C20	1.5 (5)
C1—C2—C3—C4	0.7 (5)	C17—C18—C19—Br4	−179.2 (2)
Br1—C2—C3—C4	179.6 (2)	Cu1—O2—C20—C19	177.6 (2)
C2—C3—C4—C5	0.0 (5)	Cu1—O2—C20—C15	−3.4 (4)
C2—C3—C4—Br2	−179.4 (2)	C18—C19—C20—O2	176.3 (3)
C3—C4—C5—C6	−0.3 (5)	Br4—C19—C20—O2	−3.0 (4)
Br2—C4—C5—C6	179.1 (2)	C18—C19—C20—C15	−2.8 (5)
C4—C5—C6—C1	0.0 (5)	Br4—C19—C20—C15	177.9 (2)
C4—C5—C6—C7	178.6 (3)	C16—C15—C20—O2	−176.8 (3)
O1—C1—C6—C5	179.7 (3)	C14—C15—C20—O2	3.3 (5)
C2—C1—C6—C5	0.6 (4)	C16—C15—C20—C19	2.3 (4)
O1—C1—C6—C7	1.1 (5)	C14—C15—C20—C19	−177.6 (3)
C2—C1—C6—C7	−177.9 (3)	O1—Cu1—O3—C21	94.1 (3)
C8—N1—C7—C6	177.3 (3)	O2—Cu1—O3—C21	5.0 (3)
Cu1—N1—C7—C6	−3.4 (4)	N1—Cu1—O3—C21	−172.4 (3)
C5—C6—C7—N1	177.4 (3)	N2—Cu1—O3—C21	−88.6 (3)
C1—C6—C7—N1	−4.0 (5)	Cu1—O3—C21—N3	−66.7 (4)
C7—N1—C8—C9	1.1 (5)	C23—N3—C21—O3	−179.6 (4)
Cu1—N1—C8—C9	−178.4 (2)	C22—N3—C21—O3	−4.2 (5)
C7—N1—C8—C13	−178.9 (3)	O4—C24—N4—C26	0.4 (17)
Cu1—N1—C8—C13	1.6 (3)	O4—C24—N4—C25	−176.8 (12)
C13—C8—C9—C10	0.9 (5)	O4'—C24'—N4'—C25'	177 (4)
N1—C8—C9—C10	−179.1 (3)	O4'—C24'—N4'—C26'	1(6)

### Hydrogen-bond geometry (Å, °)

**Table:**

*D*—H···*A*	*D*—H	H···*A*	*D*···*A*	*D*—H···*A*
C5—H5···O3^i^	0.95	2.47	3.319 (4)	148
C7—H7···O3^i^	0.95	2.37	3.243 (4)	153
C14—H14···O4^ii^	0.95	2.33	3.226 (14)	156
C22—H22A···O1	0.98	2.54	3.378 (4)	143

Symmetry codes: (i) −*x*+1, −*y*+1, −*z*+1; (ii) −*x*+2, −*y*+1, −*z*+2.
